# Health Benefits of Cycling as a Form of Active Travel: A Pilot Empirical Study

**DOI:** 10.3390/ijerph23010079

**Published:** 2026-01-06

**Authors:** Mehrnaz Zargarzadeh, Anabela Salgueiro Narciso Ribeiro, Amândio Manuel Cupido Santos, Rafael Nogueira Rodrigues

**Affiliations:** 1 Department of Civil Engineering, Faculty of Sciences and Technology, University of Coimbra, 3030-788 Coimbra, Portugal; anabela@dec.uc.pt; 2 Interdisciplinary Center for the Study of Human Performance (CIPER), Faculty of Sport Science and Physical Education, University of Coimbra, 3040-248 Coimbra, Portugal; amandiosantos@fcdef.uc.pt; 3 Association for the Development of Industrial Aerodynamics (ADAI), Department of Mecanic Engineering, Faculty of Sciences and Technology, University of Coimbra, 3030-790 Coimbra, Portugal; 4 University of Coimbra Stadium, University of Coimbra, Santa Clara, 3040-256 Coimbra, Portugal; rafael.rodrigues@uc.pt

**Keywords:** physical activity, active modes, health, well-being, laboratory experiments

## Abstract

**Highlights:**

**Public health relevance**

- This study examines how integrating cycling into daily routines can improve physical and mental health in an academic setting, addressing public health challenges such as physical inactivity and sedentary lifestyles.

**Public health significance**

- The pilot study demonstrated that even modest daily cycling led to significant health benefits, including reduced body fat, improved cardiovascular recovery, and enhanced aerobic capacity, particularly in university students and staff.

**Public health implications**

- These findings suggest that implementing cycling programs, such as the UCicletas initiative, in academic settings could be a sustainable and cost-effective strategy to promote physical activity and improve public health outcomes.

**Abstract:**

Integrating physical activity into daily routines through walking and cycling supports health while promoting sustainable mobility. This assumption aligns with SDGs 3, 5 and 11. This study assessed the feasibility and health impacts of cycling within a university setting. As part of the *UCicletas* program at Coimbra University, sixteen participants (8 males, 8 females) used conventional or pedal-assist bicycles for eight weeks. Descriptive analyses, *t*-tests, and Spearman correlations were applied to anthropometric and cardiorespiratory measurements collected before and after the intervention. Weekly cycling distance was obtained through self-reported odometer values. After eight weeks, notable health improvements were observed. Body fat decreased by 1.8% overall, with a significant reduction in females (*p* < 0.05). VO_2max_ increased by 13.79% in males (*p* = 0.02) and 12.21% in females (*p* = 0.03). The Ruffier Index decreased by 18.87% in males (*p* < 0.05) and 14.73% in females (*p* = 0.03). Gender differences were evident in correlations: male BMI showed a strong negative association with respiratory recovery (ρ = −0.867, *p* = 0.005), whereas the female association was weak (ρ = 0.371). Correlations between cycling distance and health outcomes were weak and non-significant. Overall, the findings confirm that modest daily cycling improves health outcomes.

## 1. Introduction

Physical inactivity impacts public health at the global level, representing a leading risk factor for noncommunicable diseases (NCDs), including cardiovascular disease, type 2 diabetes, obesity, and certain types of cancer. To mitigate these effects, the World Health Organization (WHO) recommends that adults engage in at least 75 to 150 min of vigorous aerobic exercise, 150 to 300 min of moderate aerobic exercise, or a combination of both per week [1,2,3,4].

Active travel, including walking and cycling, is one effective strategy to increase physical activity. Incorporating these activities into daily commuting allows individuals to meet recommended physical activity guidelines without needing extra time for structured exercise. Cycling is a powerful preventive strategy for many diseases [3,5,6,7]. Moderate-intensity cycling (12–16 km/h, around 36 km per week) aligns with WHO guidelines [8]. Cycling also improves mental health, reducing risks for depression, bipolar disorder, and schizophrenia [9,10,11,12].

Bicycle use for daily commuting depends on several factors. The built environment plays a critical role, including urban design, well-maintained infrastructure, secure parking, safe traffic conditions, suitable terrain, access to changing facilities or showers, proximity to diverse amenities, personal safety, green spaces and globally aesthetically pleasing environments [13,14,15,16,17,18,19,20,21,22,23,24]. The impacts of this use on health are also promoted through awareness campaigns. Recent data from the European Commission’s Eurobarometer report indicates that nearly 36% of Europeans now incorporate cycling or other physical activities into their daily commuting at least once a week, reflecting the growing recognition of cycling as an essential means of promoting health [18]. Moreover, the modal shift toward cycling rather than car trips helps reduce congestion and emissions in urban areas.

However, demographic and socioeconomic factors, such as age, education level, family structure, and income status, significantly influence active travel behaviours [20,21,22,23]. One of the few studies incorporating both built environment and individual characteristics is by Kroesen and van Wee [24], whose empirical model highlights the complex interactions between infrastructure, personal awareness, and demographic factors in shaping active travel behaviours and associated health outcomes.

In response to these challenges, many universities worldwide have launched health promotion programs to improve the well-being of students and staff through physical activity. For example, programs such as Stanford University’s *BeWell* program (https://bewell.stanford.edu/bewell-program, accessed on 18 December 2025), the Active Workplaces initiative at the University of Queensland, and the (kind)mind program at the National University of Singapore focus on integrating wellness into daily routines [25]. These initiatives have been shown to improve physical fitness and mental health among university populations by promoting physical activity and providing support for mental health challenges [26,27]. In Europe, Health Promoting University (HPU) initiatives are also integrating physical activity, mental health services, and health education into campus environments, often supported by national policies and school-based programs [28,29].

While numerous studies have explored the health benefits of walking and cycling through structured, short-term interventions in controlled or semi-supervised settings, the long-term effects of these activities as part of daily routines, particularly in academic settings, remain underexplored.

This study aims to address this gap through a pilot study in the University of Coimbra (UCicletas program), which offers bicycles at very low cost to students and staff. Participants periodically assess their physical health as they integrate cycling into their daily routines. By providing affordable access to bicycles and encouraging daily cycling, the program aims to foster long-term behaviour change and promote sustainable transport practices. Additionally, this study emphasises the importance of raising awareness of the health benefits of active travel to encourage healthier, more sustainable mobility choices.

The specific contribution of this study is a focus on gradual behaviour change, measuring health outcomes at multiple intervals (every 8 weeks) to track participants’ progress and encourage continued cycling.

## 2. Materials and Methods

### 2.1. Study Design

This study is a collaboration between the Faculty of Sport Sciences and Physical Education (FCDEF) University of Coimbra Stadium (UC Sports), and the Faculty of Sciences and Technology at the University of Coimbra, Portugal. Is part of ongoing research at the University of Coimbra (UC) that began in 2022, as part of European project “Cycling Campus & City (3Cs-Project: 101090685, ERASMUS-SPORT-2022-SCP”, https://www.uc.pt/en/3cs/, accessed on 18 December 2025), exploring how mobility habits influence health through surveys, laboratory experiments, workshops, and various projects. One initiative within this research is the *UCicletas* project (https://www.uc.pt/desporto/ucicletas/, accessed on 18 December 2025), which promotes active mobility by offering bicycles for temporary use to students, faculty, researchers, and staff.

#### 2.1.1. UCicletas Program Description

The UCicletas program offers both conventional bicycles (*n* = 10) and pedal-assist electric bicycles (pedelecs, *n* = 30), each with different usage fees. Participants could select the bicycle type that best suited their mobility needs, fitness levels, and economic considerations. Pedelecs provide motor assistance only while the user is actively pedalling, ensuring that physical effort is always involved. The inclusion of pedelecs was essential given Coimbra’s steep topography, which can limit cycling uptake, particularly among beginners or individuals with lower fitness levels. Despite motor assistance, significant physical effort is still required, as confirmed by previous research [30,31]. Pedelecs are classified as low-emission mobility modes, with life-cycle assessments showing substantially lower environmental impacts than those of private motorised vehicles [32,33]. Including pedelecs alongside conventional bicycles aligns with studies examining active and low-impact transport systems. Their integration in the program enables the assessment of cycling behaviours within mobility modes recognised as environmentally efficient in urban contexts. Pedelecs also allow participation for individuals who might otherwise be discouraged by the city’s terrain or physical limitations, while still promoting active mobility. This real-world design captures a broad spectrum of cycling behaviours and health outcomes, from those seeking to reduce exertion to those engaging in more physically demanding cycling.

#### 2.1.2. Cycling Exposure Measurement

Cycling activity was monitored using the onboard cycle computers of each e-bike, which recorded both total distance and speed as part of the study’s assessment. These devices used a speed sensor attached to the wheel, detecting each rotation as a wheel-mounted magnet passed the sensor. The signal was then transmitted to the cycle computer, which calculated both velocity and distance based on the calibrated wheel diameter. Participants self-reported their weekly total distance travelled. However, due to data access restrictions and the mobility service’s privacy protocols, automated digital extraction of cycling data was not possible. As a result, participants manually reported their odometer readings. At the final assessment, the research team manually verified all odometer readings and cross-checked them with the participants’ reports, confirming that the recorded distances matched.

#### 2.1.3. Environmental Context and Real-World Conditions

Cycling activity occurred under participants’ usual daily-life conditions, and no standardised route, duration, or intensity was prescribed. Consequently, environmental characteristics such as terrain, weather, traffic, and surface conditions were not controlled. This real-world design aligns with the natural use of the UCicletas program but introduces inherent variability in cycling exposure. Cycling volume was therefore quantified solely through the distance recorded by the onboard cycle computer, verified by weekly self-reports.

#### 2.1.4. Health Outcome Assessments

To track the effects of daily cycling on health, participants undergo laboratory assessments every eight weeks, including standardised anthropometric measurements (e.g., BMI and body composition) and cardiovascular fitness tests (e.g., Ruffier–Dickson Index), following ACSM and WHO protocols. Professor Amândio Santos conducted these measurements at the FCDEF laboratory, through a recognised professional assessment of top cycling athletes’ performances and conducted to the highest standards (https://cyclinguptodate.com/joo-almeida, accessed on 18 December 2025). These laboratory assessments provided reliable indicators of cardiovascular fitness and changes in body composition throughout the study.

**Figure 1:**
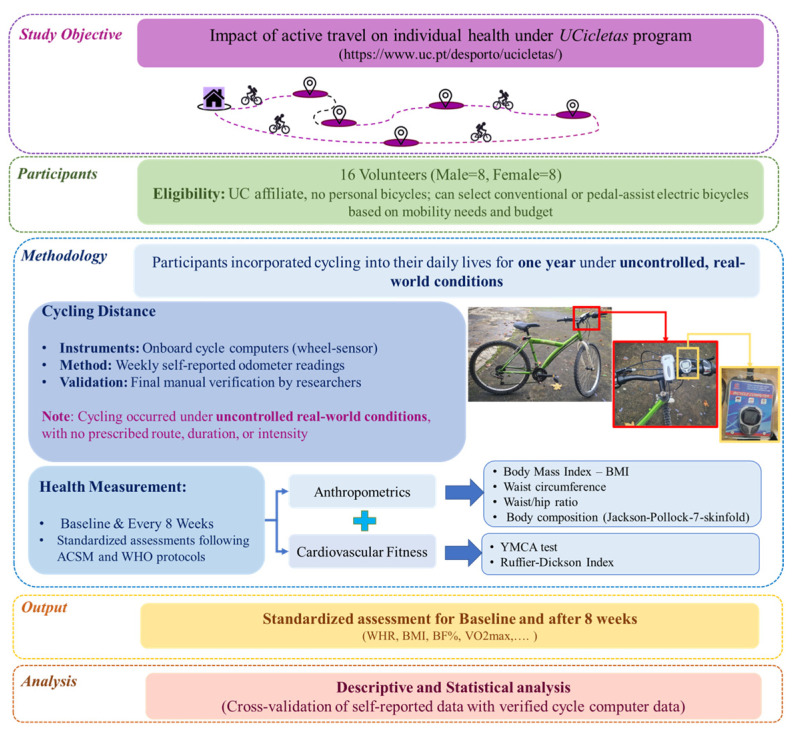
Methodological framework (photographs by Rafael Rodrigues).

The study methodology is presented in Figure 1, which illustrates the entire process. The following section describes the methods and instruments used to measure the health factors. 

### 2.2. Study Participants

Participants for this study were selected based on specific eligibility criteria. These criteria required individuals to be affiliated with the University of Coimbra (as a student, researcher, staff member, or professor) and to neither own a personal bicycle nor use one daily. A total of sixteen (16) eligible participants (eight males, eight females) were enrolled at the start of the intervention, with no withdrawals reported. The study began in 2024 and is scheduled to last for one year. Data collection and assessments were conducted between April and June. Most participants were aged 28–48 years, with females averaging 39.75 years and males averaging 36.13 years. Both groups had a comparable age distribution, with nearly identical medians for men and women, although a few outliers slightly extended the overall range. All participants provided informed consent before participating in the study and agreed to the confidential use of relevant personal information, with ethical approval obtained from the FCDEFUC Ethics Committee (Ref: CE/FCDEF-UC/00072025).

### 2.3. Anthropometrics and Cardiorespiratory Fitness Measurements and Instruments

In this study, two main categories of assessments were conducted: anthropometrics and cardiorespiratory Fitness. Both were evaluated using standardised instruments and procedures to ensure accuracy and reproducibility.

Anthropometric measurements are non-invasive assessments that provide critical information about body composition, nutritional status, disease risk, and overall health [34]. These measurements are particularly useful for identifying cardiovascular risks and enabling early intervention [35,36]. The primary indices assessed included Body Mass Index (BMI, kg/m^2^) [37], Waist Circumference (WC) [38], and Waist–Hip Ratio (WHR) [39], which together provided insight into adiposity and fat distribution.

Body height was measured to the nearest 1 mm using a calibrated stadiometer, while body weight was measured to the nearest 100 g with a beam scale (SV-Seca 710, Hamburg, Germany). Measurements were conducted under standardised conditions with participants barefoot and wearing light clothing. Waist and hip circumferences were measured using a flexible, non-elastic anthropometric tape, following World Health Organisation (WHO) guidelines. Waist circumference was measured at the midpoint between the lowest rib and the iliac crest, and hip circumference at the widest portion of the buttocks. Each measurement was taken twice, and the mean value was recorded.

Additionally, body composition was evaluated using the Jackson–Pollock 7-site skinfold (SKF) method, which involved measuring subcutaneous fat at seven standardised anatomical sites: chest, midaxillary, triceps, subscapular, abdomen, suprailium, and thigh. The assessments were performed using Harpenden skinfold callipers (Baty International, West Sussex, UK), following the ACSM (American College of Sports Medicine) Standardised Description of Skinfold Sites and Procedures to ensure accuracy and consistency [40,41]. All measurements were taken on the right side of the body, and the average of three consecutive readings was recorded for each site. The total sum of these skinfold measurements (SS) was used to determine body fat percentage (BF%), where SS represents the combined thickness (in millimetres) of the seven measured sites. BF% was calculated using specific equations distinguished by sex [42], presented in the following Equations (1) and (2):

(1) $${BF\%}_{Male}=1.112-\left(0.00043499\times SS\right)+\left(0.00000055\times {SS}^{2}\right)-(0.00028826\times age)$$

(2) $${BF\%}_{Female}=1.0970-\left(0.00046971\times SS\right)+\left(0.00000056\times {SS}^{2}\right)-(0.00012828\times age)$$

Cardiorespiratory fitness reflects the ability of the heart, lungs, and muscles to supply and utilise oxygen during sustained physical activity. VO_2max_, the most reliable indicator of aerobic capacity, is estimated through standardised methods that analyse heart rate (HR) responses to exercise intensity [43,44]. Two commonly used assessments, the Ruffier–Dickson Index (RDI) and the Indirect-Submaximal Cycle Ergometer Test (YMCA Test), provide indirect but practical measures of cardiovascular endurance [45,46].

RDI evaluates cardiovascular response by measuring heart rate at rest (HR1), immediately after performing 30 squats (HR2), and one-minute post-exercise (HR3) [47,48]. It is designed to minimise the influence of resting heart rate, with lower scores indicating better fitness levels. The Ruffier Index (RI) and Ruffier–Dickson Index (RDI) are calculated using Equations (3) and (4) [49]:


(3) $$RI=\frac{\left({HR}_{1}+{HR}_{2}+{HR}_{3}-200\right)}{10}$$

(4) $$RDI=\frac{\left(({HR}_{2}-70)+2{(HR}_{3}-{HR}_{13}\right)}{10}$$

Cardiorespiratory fitness was also evaluated using the YMCA Submaximal Cycle Ergometer Test. The test was conducted on a calibrated cycle ergometer and followed a multi-stage, submaximal protocol with workload increments based on the participant’s heart rate response. Heart rate was monitored continuously, and VO_2max_ was estimated using standardised prediction equations. The test aims for heart rate values between 110 bpm and 85% of the age-predicted maximum heart rate (220 − age) [46,50,51] and follows the ACSM guidelines for exercise testing and prescription [41] to ensure consistency in protocol and assessment.

### 2.4. Study Limitations

**Figure A1:**
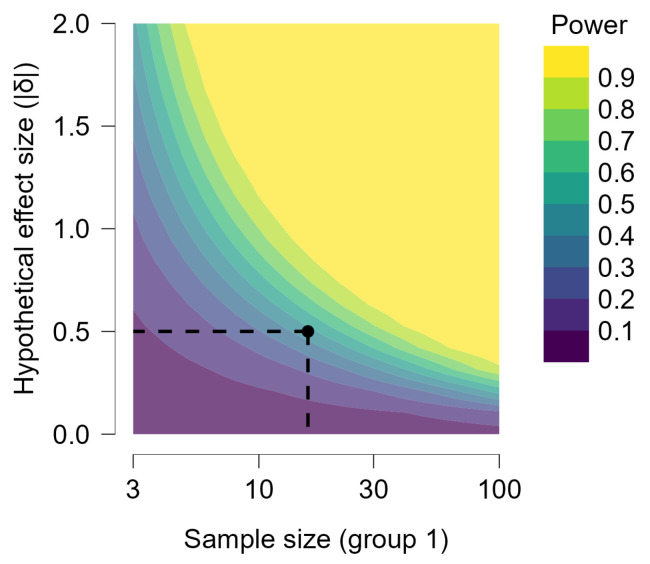
Power contour. The power contour plot shows how the sensitivity of the test changes with the hypothetical effect size and the sample sizes in the design. As we increase the sample sizes, smaller effect sizes become reliably detectable. The power contour plot shows how the sensitivity of the test changes with the hypothetical effect size and the sample sizes in the design. As we increase the sample sizes, smaller effect sizes become reliably detectable.

**Figure A2:**
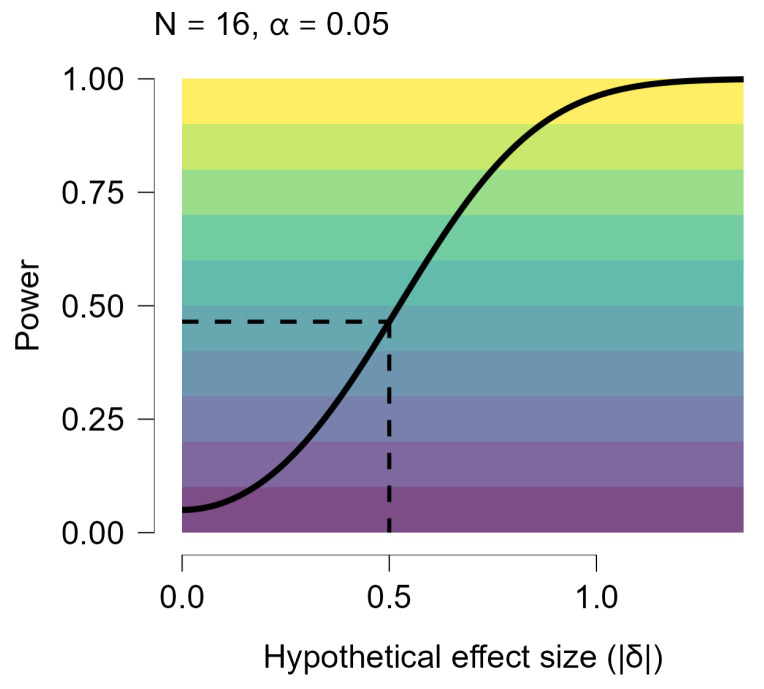
Power Curve by Effect Size. The power curve above shows how the sensitivity of the test and design is larger for larger effect sizes. If we obtained sample sizes of 16 our test and design would have power of at least 0.465 to effect sizes of |δ| > 0.5. We would be more than likely to miss (power less than 50%) effect sizes less than |δ|=0.524.

This study has several limitations. The lack of a control group prevents definitive conclusions about the intervention’s effects, as there was no direct comparison to a baseline or non-intervention group. Although the observed improvements are attributed to cycling, they could also stem from unrelated factors, such as seasonal variations, dietary changes, or reduced stress. The small sample size (*n* = 16), primarily consisting of students and staff, limits the generalizability of the findings. While this sample size provided sufficient power to detect moderate to large effect sizes, it resulted in a lower power (46.5%) for detecting more minor effects. The power analysis (see more details in the Appendix A, Table A1 and Figure A1 and Figure A2) indicated that for smaller effect sizes (below |δ| = 0.524), the likelihood of detecting significant effects drops substantially. Future studies with larger sample sizes would help increase the power and improve the ability to detect smaller effects with greater confidence. Other limitations include the one-year duration and the requirement for participants to attend lab sessions every 8 weeks may have led to decreased engagement, potentially affecting data reliability. Additionally, self-reported cycling data could introduce inaccuracies, with participants possibly overestimating trip durations or underreporting distances. Although manual verification of odometer readings was conducted by the research team at the final assessment to ensure consistency, self-reporting remains a limitation due to the potential for error. While GPS data is more objective, it may still be influenced by signal dropouts and algorithm limitations, impacting accuracy. Furthermore, because cycling occurred in uncontrolled real-world environments, variability in environmental conditions (e.g., slope, weather, surface quality, and traffic) could not be quantified, which may have influenced the physical demands experienced by participants.

The study did not capture detailed physical activity data, including cycling intensity, activity levels, exercise duration, cycling time, and calories burned. These measures would be necessary for a complete assessment of physiological workload and to better understand the relationship between cycling and health outcomes. The primary focus was on the feasibility of integrating cycling into daily routines and describing mobility behaviour in a real-world university setting, rather than on detailed physiological assessment. In addition, sociodemographic and health-related data (e.g., physical activity history, smoking, alcohol consumption, and diet) were not collected, limiting the ability to examine individual determinants of cycling behaviour and their effects on health outcomes.

To address these gaps, future research will incorporate a more comprehensive sociodemographic and health questionnaire alongside detailed physical activity measures, including cycling intensity, duration, and calories burned, to provide a more complete assessment of the physiological and health impacts of cycling.

## 3. Results

### 3.1. Cycling Distance by Age and Gender

As mentioned in the previous section (see Section 2.1), cycling distance was measured by self-reporting. The total distance cycled by age and gender is presented in Table 1, with detailed weekly cycling data in the Appendix A, Table A2. For younger adults (<35 years), the total distance cycled was 1933.80 km, with females covering 1165.39 km and males 768.41 km. For older adults (>35 years), the total distance cycled was 1661.88 km, with females cycling 737.67 km and males cycling 924.21 km.

### 3.2. Descriptive Statistical Results

According to the lab protocols outlined in the previous section, health parameters were measured for all 16 participants. Table 2 summarises the primary health parameters at baseline, their values after 8 weeks of cycling, and the observed changes during this period. The detailed measured parameters are provided in the Appendix A (Table A3 and Table A4). Data analyses were performed using JASP (Version 0.19.3) (https://jasp-stats.org/, accessed on 20 December 2025) (open-source software) and Microsoft Excel.

After eight weeks of cycling intervention, modest changes were observed in several health parameters, particularly in BMI and %BF. More pronounced improvements were noted in cardiorespiratory fitness, as evidenced by favourable changes in VO_2max_ and cardiovascular recovery indices. RI decreased by 18.87% in males (from 10.65 to 8.64) and by 14.73% in females (from 9.71 to 8.28), indicating improved cardiovascular efficiency. Similarly, RID decreased by 22.96% in males (from 9.54 to 7.35), while females exhibited a slight increase of 3.69% (from 7.85 to 8.14). The estimated VO_2max_ increased by approximately 14% in males and 12% in females. In terms of absolute VO_2max_ values, males demonstrated an increase from 2.14 to 2.38 L·min^−1^ (11.21%), whereas females improved from 1.53 to 1.72 L·min^−1^ (12.42%).

### 3.3. Statistical Tests and Regressions

#### 3.3.1. *t*-Test

The paired *t*-test, presented in Table 3 (see more details in the Appendix A, Table A5), compare key health and fitness parameters before and after the 8-week cycling. The null hypothesis, which stated that the intervention would not lead to significant improvements, was rejected. The paired *t*-test was applied based on the assumption of normality, confirmed by the Shapiro–Wilk test. Normality was upheld for almost all parameters (except WHR for both gender) in both the male and female groups (*p* > 0.05). Therefore, there was no need to add a non-parametric test. The Shapiro–Wilk test value including w and *p*-values obtained were included in Table 3. The findings indicate notable improvements in body composition and cardiorespiratory fitness, with some gender differences. Table 3. Paired *t*-test and Shapiro–Wilk test results comparing baseline and after 8 weeks cycling.

After 8 weeks of cycling, significant changes were observed in the total sample. Weight (MD = −0.71 kg, t = −2.90, *p* = 0.011) and BMI (MD = −0.25 kg/m^2^, t = −2.68, *p* = 0.017) significantly decreased. Males showed significant reductions in weight (MD = −0.79 kg, t = −3.07, *p* = 0.02) and BMI (MD = −0.26 kg/m^2^, t = −2.89, *p* = 0.02), while females showed no significant changes in either variable. The total sample exhibited a significant decrease in the RI (MD = −1.73, t = −4.62, *p* < 0.001). Males showed a significant reduction in the RDI (MD = −2.19, t = −4.55, *p* < 0.001). Estimated VO_2max_ increased significantly in both males (MD = 3.65 mL·kg^−1^·min^−1^, t = 3.19, *p* = 0.02) and females (MD = 2.94 mL·kg^−1^·min^−1^, t = 2.65, *p* = 0.03). Absolute VO_2max_ also showed significant improvement in the total sample (MD = 0.22 L/min, t = 3.86, *p* < 0.001) and in males (MD = 0.25 L/min, t = 3.15, *p* = 0.02).

#### 3.3.2. Spearman Correlation: Health Changes and Cycling Distance

This study examines the Spearman correlations between changes in health parameters (DF: 8-week results minus baseline) and total cycling distance across all participants (Table 4). The Pearson correlation coefficients, organised by gender (females and males) and age groups (younger adults: <35 years; older adults: >35 years), are provided in the Appendix A (Table A6, Table A7, Table A8 and Table A9). These analyses provide a comprehensive understanding of how body composition, aerobic fitness, and endurance performance are interrelated.

Overall, the correlations among the factors for all participants ranged from weak to moderate, both positive and negative. Weak negative correlations were found between DF-Weight, DF-BMI, and DF-WHR with DF-RI (Spearman’s rho (ρ) = −0.153, −0.144, and −0.148, respectively), and between DF-Weight and DF-BMI with DF-ESVO2max (ρ = −0.295 and −0.278, respectively). DF-BF% showed a weak negative correlation with DF-ESVO2max (ρ = −0.052) and a moderate positive correlation with DF-RI (ρ = 0.394), although neither of these correlations was statistically significant. Significant correlations were observed between DF-Weight and DF-BMI (ρ = 0.998, *p* < 0.001), as well as between DF-ESVO2max and DF-ABVO2max (ρ = 0.973, *p* < 0.001).

A gender- and age-based analysis revealed significant differences. DF-Weight and DF-BMI were strongly correlated in both females (ρ = 0.994, *p* < 0.001) and males (ρ = 0.994, *p* < 0.001), confirming their close association. This strong correlation suggests a consistent relationship between body weight and BMI across both sexes. However, the relationship between DF-BMI and respiratory function differed between sexes. In females, DF-BMI showed a weak positive correlation with DF-RI (ρ = 0.371), while in males, the correlation was strong and negative (ρ = −0.867, *p* = 0.005). This difference suggests that increased BMI may have a more pronounced negative impact on respiratory recovery in males. For DF-BF%, females exhibited a moderate positive correlation with DF-RDI (ρ = 0.539), whereas the correlation was weaker in males (ρ = 0.404). This indicates that body fat percentage may play a stronger role in cardiovascular recovery in females compared to males. Both sexes showed a negative correlation between DF-BF% and DF-ESVO, suggesting that higher body fat negatively affects oxygen utilisation efficiency, although these correlations were not statistically significant (females: ρ = −0.095, males: ρ = −0.156). This consistent but non-significant relationship across both genders implies a potential, though weak, effect of body fat on oxygen efficiency.

The correlations between cycling distance and various health parameters were weak and non-significant in both males and females. For DF-Weight, males exhibited a weak positive correlation (ρ = 0.349, *p* = 0.396), while females showed a weak negative correlation (ρ = −0.491, *p* = 0.217). In the case of DF-BMI, males had a weak positive correlation (ρ = 0.287, *p* = 0.49), whereas females displayed a weak negative correlation (ρ = −0.476, *p* = 0.243). For DF-BF%, both males and females exhibited negative correlations, with males showing ρ= −0.240 (*p* = 0.568) and females ρ = −0.548 (*p* = 0.171). The DF-RI correlation with cycling distance was very weak for both sexes, with males at ρ = −0.036 (*p* = 0.933) and females at ρ = −0.551 (*p* = 0.157). Similarly, DF-RDI showed weak negative correlations, with males at ρ = −0.262 (*p* = 0.536) and females at ρ = −0.551 (*p* = 0.157). DF-ESVO_2_ and DF-ABVO_2_ showed very weak correlations, with males at ρ = −0.071 (*p* = 0.882) and females at ρ = 0.071 (*p* = 0.882) for DF-ESVO_2_, and males at ρ = −0.238 (*p* = 0.582) and females at ρ = −0.214 (*p* = 0.619) for DF-ABVO_2_.

Age-related trends were also observed. In younger adults (<35 years), both DF-Weight and DF-BMI were strongly negatively correlated with DF-RI (ρ = −0.844 and −0.847, respectively). In older adults (>35 years), these correlations weakened and became non-significant (ρ = 0.253 and 0.264).

Lastly, DF-WHR showed a moderate positive correlation with total distance in older adults (ρ = 0.575, *p* = 0.105). DF-RI exhibited a stronger negative correlation with total distance in females (ρ = −0.551), while the relationship was weaker in males (ρ = −0.036).

## 4. Discussion

This study aimed to evaluate the effects of an 8-week cycling intervention on body composition, aerobic capacity, and cardiovascular recovery in a cohort of 16 university students and staff. The first important aspect of this study’s results is that the discussion of the results must be cautious and validated by further studies. The intervention led to modest improvements in body composition, with reductions in weight and BMI observed, particularly in males. These changes were statistically significant for males but not for females. However, gender, age, and baseline fitness levels influenced the extent of these changes. Weight loss was observed in half of the participants, while a few experienced minimal weight gains. These outcomes are consistent with studies such as Naruse et al. (2023) [52], who demonstrated that cycling induces muscle development (hypertrophy), which may partly explain the minimal weight gain observed in some participants. Furthermore, Rhee (2017) [53] notes that weight cycling, the process of repeatedly losing and regaining weight, can cause fluctuations in body composition, including muscle gain, even with overall weight loss. Additionally, Betz et al. (2025) [54], found that aerobic exercise, like cycling, enhances muscle capillarization, improving muscle efficiency and potentially contributing to muscle growth.

Moreover, the cycling intervention resulted in reductions in body fat percentage (BF%) for both males and females, although the magnitude of these changes varied by gender. Males showed more significant decreases in BF%, consistent with previous studies suggesting that males generally experience greater fat loss due to higher muscle mass and more efficient fat oxidation [55]. The observed gender differences in body composition and cardiovascular recovery outcomes may be due to both physiological and behavioural factors, including baseline fitness levels, muscle mass, and adherence to the intervention. While females generally showed less pronounced improvements, they still experienced health benefits, particularly in cardiovascular recovery [56]. Testosterone levels in males further support this by enhancing fat metabolism and preserving lean muscle mass [49]. For females, although fat loss was less pronounced, there were notable improvements in waist-to-hip ratio (WHR), suggesting favourable fat redistribution, particularly around the abdominal region, which is vital for reducing metabolic risk. This pattern is consistent with evidence showing that central fat redistribution plays a key role in mitigating metabolic syndrome risk [57]. The cycling intervention led to improvements in cardiovascular recovery, as reflected by reductions in both the Ruffier Index (RI) and Ruffier–Dickson Index (RDI). While males exhibited more substantial improvements, females also showed positive, albeit more minor, changes in these indices, reflecting apparent sex-specific differences in autonomic function and cardiovascular adaptation to aerobic training [58,59]. These differences may stem from greater stroke volume, higher baseline aerobic capacity, and more efficient parasympathetic reactivation in males, which results in better heart-rate recovery after submaximal exercise.

Overall, Tobia et al. (2025) [25] provide further evidence that employees engaging in workplace exercise sessions experienced comparable reductions in blood pressure and cardiovascular risk, reinforcing the view that regular aerobic activity improves autonomic and cardiovascular regulation.

Both males and females showed moderate improvements in aerobic capacity, with significant increases in estimated and absolute VO_2max_ values, particularly for males. However, males showed greater increases in VO_2max_, consistent with previous findings. Comparable sex-specific differences in aerobic adaptations have been reported in university-based training programs, with males typically demonstrating larger VO_2max_ gains than females [56]. These differences may reflect males’ greater muscle mass, cardiovascular efficiency, and endurance performance capacity [60,61]. Females, demonstrated slower recovery times, which may be attributed to hormonal differences or lower baseline fitness levels [62]. Notably, increased body fat (as measured by BMI) was associated with poorer recovery, particularly in males. The negative correlation between BMI and the recovery index (RI) in males underscores the role of excess weight in impairing recovery [63,64]. University-based aerobic interventions have similarly shown that improvements in metabolic and cardiovascular function can occur even when BMI changes are modest, reinforcing the modest BMI reductions observed in our cohort [56]. These findings highlight that fat loss, rather than BMI reduction alone, contributes more meaningfully to improved cardiovascular recovery [65]. In females, although improvements in VO_2max_ were less pronounced than in males, the cycling intervention still resulted in significant cardiovascular benefits, including reductions in systolic blood pressure and improved cholesterol levels [66,67]. This aligns with our study’s findings, which showed that females showed improved cardiovascular recovery even in the absence of substantial VO_2max_ gains. Tobia et al. (2025) [25] similarly report reductions in systolic and diastolic blood pressure after workplace exercise, supporting the idea that even moderate-intensity programs enhance cardiovascular health regardless of changes in VO_2max_.

Age also influenced the outcomes of the cycling intervention. Younger adults (under 35 years) showed more significant improvements in both body composition and aerobic capacity. Similar patterns have been documented among competitive cyclists, where senior athletes exhibit higher VO_2max_ values, greater long-duration power, and better fatigue resistance than younger riders, suggesting that age influences endurance capacity even in trained populations [68]. These differences may be partly explained by greater metabolic efficiency and better muscle mass preservation among younger adults [64]. In contrast, older adults (over 35 years) demonstrated less pronounced changes in body composition and aerobic capacity, likely due to age-related declines in fat metabolism and muscle mass [63,69]. Despite these limitations, older adults still showed improvements in cardiovascular recovery, indicating that cycling provides cardiovascular benefits across age groups. This is further supported by findings from Tegegne et al. (2025) [70], who found that cycling interventions significantly improve cardiovascular health parameters in older adults. These benefits may be due to enhanced vascular endothelial function, improved heart rate variability (HRV), and better regulation of blood pressure [67].

The effects of age and gender on cycling performance were also significant. Younger adults cycled longer distances than older adults, as expected, since younger individuals typically have higher endurance capacity [71,72]. This trend is further supported by evidence from national-level cyclists, where senior athletes demonstrate superior long-duration power outputs and enhanced performance during prolonged efforts compared with juniors, indicating that endurance-related capacities continue to mature with age even among trained individuals [68]. Within the older subgroup, females cycled longer distances than age-matched males. Although motivational or adherence-related factors may contribute to this pattern [73], university-based interventions also report gender-specific responses, with females consistently adhering to exercise protocols and achieving meaningful, although sometimes more minor, improvements in body composition and performance [56]. These findings suggest that gender-related behavioural and physiological factors influence training outcomes and support the alignment between our results and prior work in university and athletic populations.

## 5. Conclusions and Future Research

This study investigated the impact of integrating cycling into daily routines on anthropometric measures and cardiorespiratory fitness over 8 weeks with 16 participants aged 19–56. Unlike many interventions conducted in controlled or academic settings, this study focused on cycling in daily life, making it highly relevant to real-world applications. The results indicate modest improvements in several health parameters, including weight loss and reduced body fat percentage. While these changes were generally consistent across both males and females, no significant improvements in cardiovascular fitness were observed, which may be attributed to the short duration of the intervention

The findings suggest potential benefits of incorporating cycling into daily life, but the interpretation of these results should be cautious due to the study’s limitations. The lack of a control group and the relatively small sample size (*n* = 16) limit the ability to draw definitive conclusions about the causal effects of cycling on health outcomes. Additionally, environmental factors, such as weather and terrain, were not controlled for and could have influenced both the participants’ physical activity levels and the observed results. Furthermore, while the self-reported cycling distance was verified, it remains a potential source of error, and more accurate tracking methods could provide more reliable data in future studies.

Given the limitations of this pilot study, future research should aim to extend the intervention period and include larger, more diverse populations to better assess the long-term effects of cycling on health outcomes. Incorporating objective measures of cycling intensity and accounting for environmental factors (e.g., weather, terrain) would provide a more comprehensive understanding of cycling’s physiological impact. Additionally, exploring complementary interventions, such as behavioural coaching or nutritional guidance, could enhance the effectiveness of cycling-based health interventions.

Overall, this study demonstrates promising results, showing that even modest lifestyle changes, such as incorporating a few kilometres of cycling into daily commuting can lead to substantial health benefits. These findings underscore cycling as a practical, accessible, and sustainable approach to improving public health. By building on these results, future research can solidify cycling’s role as a key component of long-term health improvement strategies.

## Figures and Tables

**Table 1:** Distance Cycled and Percentage of Total Distance by Age Group and Gender.

Age/Gender	Female		Male	
	Total Distance (km)	% of Total Distance	Total Distance (km)	% of Total Distance
Age < 35	1165.39	32.41%	768.41	21.37%
Age > 35	737.67	20.52%	924.21	25.70%

**Table 2:** Descriptive Analysis of Health Parameters Before and After 8 Weeks of Cycling.

Health Parameters	Male (*n* = 8)			Female (*n* = 8)		
	Mean ± SD		% Changes	Mean ± SD		% Changes
	Before 8 Weeks	After 8 Weeks		Before 8 Weeks	After 8 Weeks	
Weight (kg)	81.69 ± 14.35	80.90 ± 14.45	−0.97	64.64 ± 10.28	64.00 ± 10.39	−0.99
BMI (kg/m^2^)	26.64 ± 3.84	26.39 ± 3.90	−0.94	24.44 ± 4.14	24.20 ± 4.17	−0.98
WHR	0.90 ± 0.04	0.90 ± 0.04	0	0.88 ± 0.24	0.87 ± 0.24	−1.14
% BF	24.09 ± 6.19	23.72 ± 5.74	−1.54	32.75 ± 6.82	32.11 ± 6.88	−1.95
RI	10.65 ± 4.16	8.64 ± 4.15	**−18.87**	9.71 ± 2.77	8.28 ± 2.20	**−14.73**
RID	9.54 ± 2.77	7.35 ± 3.29	**−22.96**	7.85 ± 1.54	8.14 ± 1.68	3.69
Estimated VO_2max_ (mL·kg·min^−1^)	26.47 ± 4.73	30.12 ±6.04	**13.79**	24.08 ± 5.28	27.02 ± 4.44	**12.21**
Absolute VO_2max_ (L/min)	2.14 ± 0.40	2.38 ± 0.37	**11.21**	1.53 ± 0.27	1.72 ± 031	**12.42**

**BMI**: Body Mass Index, **WHR**: Waist/Hip Ratio, **% BF**: Body Fat percentage, **RI**: Ruffier Index, **RID**: Ruffier–Dickson Index, **Estimated VO_2max_**: Maximal oxygen consumption, **Absolute VO_2max_**: Absolute maximal oxygen consumption, **SD**: standard deviation, **% Change**: the percentage difference between the before and after 8 weeks measurements. It is calculated by subtracting the baseline value (before 8 weeks) from the value after 8 weeks, dividing by the baseline value, and multiplying by 100. Red = Decrease, Green = Increase, **Bold values** indicate the greatest changes.

**Table 3:** Paired *t*-test results comparing baseline and after 8 weeks cycling.

Parameter (After 8 Weeks—Baseline)	*t*-Test (t, *p*)		Shapiro–Wilk Test (w, *p*)	
	Male (*n* = 8)	Female (*n* = 8)	Male (*n* = 8)	Female (*n* = 8)
Weight (kg)	−3.07, 0.02 *	−1.46, 0.19	0.948, 0.692	0.923, 0.453
BMI (kg/m^2^)	−2.89, 0.02 *	−1.41, 0.20	0.946, 0.674	0.918, 0.413
WHR	−0.92, 0.39	−1.21, 0.26	0.741, 0.006 **	0.806, 0.033 **
% BF	−1.47, 0.18	−2.03, 0.08 *	0.915, 0.39	0.972, 0.916
RI	−3.92, 0.01 *	−2.58, 0.04 *	0.945, 0.664	0.902, 0.304
RID	−4.55, 0.00 *	0.93, 0.38	0.960, 0.81	0.928, 0.5
Estimated VO_2max_ (mL/kg/min)	3.19, 0.02 *	2.65, 0.03 *	0.941, 0.625	0.957, 0.783
Absolute VO_2max_ (L/min)	3.15, 0.02 *	2.22, 0.06 *	0.963, 0.842	0.923, 0.453

**BMI**: Body Mass Index, **WHR**: Waist/Hip Ratio, **% BF**: Body Fat percentage, **RI**: Ruffier Index, **RID**: Ruffier–Dickson Index, **Estimated VO_2max_**: Maximal oxygen consumption, **Absolute VO_2max_:** Absolute maximal oxygen consumption, *t*-test: Paired *t*-test (Student), **t** = t-value from paired *t*-test, ***p*** = *p*-value from paired *t*-test, Shapiro–Wilk test (normality check), **W** = W-value from Shapiro–Wilk test, ***p*** = *p*-value from the Shapiro–Wilk test (used to assess the normality of the data distribution), * Indicate statistical significance (*p* < 0.05), ** Indicate a non-normal distribution.

**Table 4:** Spearman’s correlation—changes in health parameters and cycled distance of male and female participants.

Health Parameters Changes		DF-Weight		DF-BMI		DF-WHR		DF-BF%		DF-RI		DF-RDI		DF-ESVO2		DF-ABVO2		Total Distance (m)	
		Male	Female	Male	Female	Male	Female	Male	Female	Male	Female	Male	Female	Male	Female	Male	Female	Male	Female
DF-Weight	ρ	—	—																
	*p*-value	—	—																
DF-BMI	ρ	0.994 ***	0.994 ***	—	—														
	*p*-value	<0.001	<0.001	—	—														
DF-WHR	ρ	0.239	0.58	0.218	0.577	—	—												
	*p*-value	0.568	0.131	0.603	0.134	—	—												
DF-BF%	ρ	−0.109	0.275	−0.12	0.31	−0.315	0.086	—	—										
	*p*-value	0.797	0.509	0.776	0.462	0.448	0.84	—	—										
DF-RI	ρ	−0.83 **	0.319	−0.867 **	0.371	−0.424	0.031	0.404	0.731 *	—	—								
	*p*-value	0.011	0.441	0.005	0.365	0.295	0.942	0.321	0.04	—	—								
DF-RDI	ρ	−0.337	0.036	−0.371	0.036	−0.383	−0.111	0.168	0.539	0.491	−0.078	—	—						
	*p*-value	0.414	0.932	0.365	0.933	0.349	0.793	0.691	0.168	0.217	0.854	—	—						
DF-ESVO2	ρ	−0.554	−0.12	−0.587	−0.048	0.038	−0.049	−0.156	−0.095	0.563	−0.12	0.714	0.072	—	—				
	*p*-value	0.154	0.778	0.126	0.935	0.928	0.908	0.713	0.84	0.146	0.778	0.058	0.866	—	—				
DF-ABVO2	ρ	−0.639	0.072	−0.659	0.143	0.038	0.049	−0.12	0.143	0.563	0.084	0.738 *	0.144	0.976 **	0.952 **	—	—		
	*p*-value	0.088	0.866	0.076	0.752	0.928	0.908	0.778	0.752	0.146	0.844	0.046	0.734	<0.001	0.001	—	—		
Total Distance (m)	ρ	0.349	−0.491	0.287	−0.476	0.179	−0.282	−0.24	−0.548	−0.036	−0.551	−0.262	−0.036	−0.071	0.071	−0.238	−0.214	—	—
	*p*-value	0.396	0.217	0.49	0.243	0.672	0.498	0.568	0.171	0.933	0.157	0.536	0.933	0.882	0.882	0.582	0.619	—	—

* *p* < 0.05, ** *p* < 0.01, *** *p* < 0.001, DF: Difference (Result after 8 weeks minus result of baseline), ρ: Spearman correlation.

## Data Availability

The authors confirm that the data supporting the findings of this study are available within the article as an Appendix A.

## Abbreviations

The following abbreviations are used in this manuscript:

SDGs	Sustainable Development Goals
FCDEF	Faculty of Sport Sciences and Physical Education
NCDs	Noncommunicable Diseases
WHO	World Health Organization
CRF	Cardiorespiratory Fitness
VO_2max_	Maximal oxygen consumption
VO2R	Oxygen Consumption Reserve
HRR	Heart Rate Reserve
HRmax	Maximal Heart Rate
METs	Metabolic Equivalents
BMI	Body Mass Index
WC	Waist Circumference
WHR	Waist–Hip Ratio
SKF	Jackson–Pollock 7-site skinfold
ACSM	American College of Sports Medicine
BF%	Body Fat percentage
SS	Sum of these skinfold measurements
HR	Heart Rate
RDI	Ruffier–Dickson Index
RI	Ruffier Index
YMCA	Indirect-Submaximal Cycle Ergometer
SD	Standard Deviation
MD	Mean Difference
DF	Difference between Baseline and after 8 weeks

## Appendix A

**Table A1:** Power analysis result.

Power	User Defined		
	N	Cohen’s |δ|	α
0.465	16	0.500	0.050

**Table A2:** Cycling distances travelled 8 weeks.

Nr. of Volunteer	Gender	Age	Week 1	Week 2	Week 3	Week 4	Week 5	Week 6	Week 7	Week 8	Total Distance (m)
**1**	male	56	23,412	22,568	28,659	29,304	22,521	23,876	25,677	24,900	200,917
**2**	male	33	3689	9231	4433	5651	38,874	4070	9203	3550	78,701
**3**	male	37	34,689	39,231	40,433	35,651	38,874	44,070	32,920	35,560	301,428
**4**	female	31	48,907	41,320	43,753	45,152	43,800	41,198	42,100	44,930	351,160
**5**	female	56	14,691	9312	8344	9691	13,874	14,212	9203	3550	82,877
**6**	female	42	14,917	15,165	13,962	16,007	14,784	13,977	15,993	17,352	122,157
**7**	male	32	22,875	23,204	24,124	22,135	22,785	22,661	22,940	23,007	183,731
**8**	male	37	38,740	40,433	41,030	39,031	32,209	35,065	35,156	34,166	295,830
**9**	male	28	34,189	38,313	38,433	34,651	37,774	47,033	34,920	39,560	304,873
**10**	male	19	29,403	28,956	23,678	23,450	22,570	24,840	25,670	22,534	201,101
**11**	Female	46	13,574	15,574	13,987	13,022	14,376	15,200	13,955	14,480	114,168
**12**	female	30	63,240	66,720	65,217	68,172	61,298	65,773	64,890	66,270	521,580
**13**	female	31	32,190	33,138	35,355	36,514	34,970	37,320	39,209	43,960	292,656
**14**	male	47	16,007	17,872	14,917	16,807	14,784	12,980	14,793	17,872	126,032
**15**	female	43	15,574	16,099	13,574	17,872	16,107	14,793	17,600	16,700	128,319
**16**	female	39	35,651	34,699	38,800	39,231	32,200	35,160	33,921	40,488	290,150

**Table A3:** Descriptive analysis of anthropometric measures—Baseline.

Parameters	Total (*n* = 16)			Female (*n* = 8)			Male (*n* = 8)		
	Mean ± SD	95% CI Mean (Lower–Upper)	Min–Max	Mean ± SD	95% CI Mean (Lower–Upper)	Min–Max	Mean ± SD	95% CI Mean (Lower–Upper)	Min–Max
**Age**	37.94 ± 10.07	32.57–43.30	19–56	39.75 ± 9.00	32.22–47.28	30–56	36.13 ± 11.34	26.64–45.61	19.00–56.00
**Height (m)**	1.69 ± 0.08	1.65–1.73	1.57–1.88	1.63 ± 0.04	1.60–1.66	1.57–1.67	1.75 ± 0.07	1.69–1.81	1.66–1.88
**Weight (kg)**	73.16 ± 14.93	65.21–81.12	55–110.1	64.64 ± 10.28	56.05–73.23	55–79.9	81.69 ± 14.35	69.69–93.68	62.50–110.10
**BMI**	25.54 ± 4.02	23.40–27.68	19.85–34.59	24.44 ± 4.14	20.98–27.90	19.85–31.17	26.64 ± 3.84	23.43–29.85	21.27–34.59
**Waist (cm)**	84.86 ± 14.17	77.31–92.41	68–111.5	81.04 ± 18.01	65.98–96.10	68–111.5	88.69 ± 8.51	81.58–95.80	71.00–99.50
**Hip (cm)**	95.69 ± 8.10	91.38–100.01	82.6–112	93.26 ± 8.19	86.41–100.11	82.6–108	98.13 ± 7.74	91.66–104.59	85.00–112.00
**Waist/Hip ratio**	0.89 ± 0.17	0.80–0.98	0.72–1.31	0.88 ± 0.24	0.68–1.08	0.72–1.31	0.90 ± 0.04	0.88–0.93	0.84–0.95
**Triceps (mm)**	26.78 ± 11.04	20.90–32.66	16,224.00	32.38 ± 8.60	25.18–39.57	22–44	21.19 ± 10.74	12.21–30.17	6.00–38.50
**Chest (mm)**	14.25 ± 6.77	10.64–17.86	45,837.00	9.75 ± 3.01	7.23–12.27	45,824.00	18.75 ± 6.54	13.28–24.22	10.00–29.00
**MidAxilla (mm)**	22.06 ± 7.71	17.96–26.17	13,424.00	21.63 ± 8.75	14.31–28.94	13,485.00	22.50 ± 7.09	16.57–28.43	10.00–32.00
**Subscapula (mm)**	26.31 ± 8.56	21.75–30.87	14–39	26.25 ± 9.07	18.67–33.83	18–39	26.38 ± 8.65	19.14–33.61	14.00–36.00
**Suprailiac (mm)**	26.44 ± 9.16	21.56–31.32	15,189.00	26.00 ± 8.44	18.95–33.05	18–36	26.88 ± 10.40	18.18–35.57	8.00–41.00
**Abdomen (mm)**	33.50 ± 9.77	28.29–38.71	20–48	33.44 ± 11.34	23.95–42.92	20–48	33.56 ± 8.72	26.28–40.85	22.00–46.00
**Thigh (mm)**	30.00 ± 11.35	23.95–36.05	8.5–45	35.69 ± 8.29	28.76–42.61	25–45	24.31 ± 11.56	14.65–33.98	8.50–44.00
**% Body Fat**	28.42 ± 7.72	24.31–32.53	14.67–40.40	32.75 ± 6.82	27.05–38.45	25.69–40.40	24.09 ± 6.19	18.92–29.27	14.67–31.70
**Fat Mass (kg)**	21.00 ± 7.73	16.88–25.12	9.17–34.51	21.72 ± 7.75	15.24–28.19	14.21–32.22	20.28 ± 8.18	13.44–27.13	9.17–34.51
**Fat-Free Mass (kg)**	52.16 ± 11.01	46.30–58.03	38.41–75.59	42.92 ± 3.39	40.09–45.76	38.41–47.68	61.41 ± 7.29	55.31–67.50	53.33–75.59
**HRrest (bpm)**	79.75 ± 10.96	73.91–85.59	65–99	80.25 ± 9.93	71.95–88.55	65–95	79.25 ± 12.58	68.73–89.77	66.00–99.00
**HRpost (bpm)**	127.69 ± 13.17	120.67–134.71	105–160	124.75 ± 9.57	116.75–132.75	105–134	130.63 ± 16.14	117.13–144.12	112.00–160.00
**HR1min (bpm)**	94.38 ± 14.36	86.72–102.03	71–131	92.13 ± 12.47	81.70–102.55	71–111	96.63 ± 16.58	82.77–110.49	80.00–131.00
**Ruffier Index**	10.18 ± 3.45	8.35–12.02	6.2–19	9.71 ± 2.77	7.40–12.02	6.2–13.6	10.65 ± 4.16	7.17–14.13	6.30–19.00
**Ruffier–Dickson Index**	8.69 ± 2.33	7.45–9.94	6.3–15.4	7.85 ± 1.54	6.57–9.13	6.3–10.6	9.54 ± 2.77	7.22–11.85	6.80–15.40
**Estimated VO_2max_ (mL·kg·min^−1^)**	25.28 ± 5.00	22.62–27.94	17.52–32.43	24.08 ± 5.28	19.67–28.50	17.52–31.22	26.47 ± 4.73	22.52–30.43	18.13–32.43
**Absolute VO_2max_ (L/min)**	1.83 ± 0.45	1.59–2.07	1.13–2.66	1.53 ± 0.27	1.30–1.75	1.13–2.05	2.14 ± 0.40	1.80–2.47	1.62–2.66

**Table A4:** Descriptive analysis of anthropometric measures—After 8 Weeks Cycling.

Parameters	Total (*n* = 16)			Female (*n* = 8)			Male (*n* = 8)		
	Mean ± SD	95% CI Mean (Lower–Upper)	Min–Max	Mean ± SD	95% CI Mean (Lower–Upper)	Min–Max	Mean ± SD	95% CI Mean (Lower–Upper)	Min–Max
**Age**	37.94 ± 10.07	32.57–43.30	19–56	39.75 ± 9.004	32.222–47.278	30–56	36.13 ± 11.34	26.64–45.61	19–56
**Height (m)**	1.69 ± 0.08	1.65–1.73	1.57–1.88	1.628 ± 0.037	1.597–1.659	1.567–1.67	1.75 ± 0.07	1.69–1.81	1.66–1.88
**Weight (kg)**	72.45 ± 14.96	64.48–80.42	53.7–109.8	64 ± 10.391	55.313–72.687	53.7–80.8	80.90 ± 14.45	68.82–92.98	62–109.8
**BMI**	25.29 ± 4.06	23.13–27.46	19.82–34.5	24.2 ± 4.167	20.717–27.683	19.82–31.52	26.39 ± 3.90	23.13–29.65	21.1–34.5
**Waist (cm)**	83.88 ± 14.15	76.33–91.42	67–113	80.5 ± 18.323	65.182–95.818	67–113	87.25 ± 8.21	80.39–94.11	70–98
**Hip (cm)**	95.63 ± 8.22	91.24–100.01	82–109.5	93.813 ± 8.332	86.847–100.778	82–107	97.44 ± 8.24	90.55–104.33	85–109.5
**Waist/Hip ratio**	0.88 ± 0.17	0.79–0.97	0.66–1.29	0.87 ± 0.241	0.668–1.072	0.66–1.29	0.90 ± 0.05	0.86–0.93	0.82–0.96
**Triceps (mm)**	25.94 ± 10.16	20.52–31.35	15,888.00	31.75 ± 8.515	24.632–38.868	22–43	20.13 ± 8.46	13.05–27.20	12,601.00
**Chest (mm)**	14.19 ± 7.07	10.42–17.96	45,836.00	9.5 ± 2.777	7.178–11.822	45,823.00	18.88 ± 7.02	13.01–24.74	45,958.00
**MidAxilla (mm)**	21.44 ± 8.21	17.07–25.81	13,028.00	20.875 ± 8.593	13.691–28.059	12,754.00	22.00 ± 8.35	15.02–28.98	13,028.00
**Subscapula (mm)**	25.56 ± 7.83	21.39–29.74	14–37	25 ± 9.289	17.234–32.766	15–37	26.13 ± 6.66	20.55–31.70	14–33
**Suprailiac (mm)**	26.56 ± 8.69	21.93–31.19	15,311.00	26.25 ± 8.362	19.259–33.241	16–36	26.88 ± 9.57	18.88–34.87	15,311.00
**Abdomen (mm)**	32.28 ± 8.39	27.81–36.75	21–46	33 ± 10.474	24.243–41.757	21–46	31.56 ± 6.32	26.28–36.85	21–40
**Thigh (mm)**	28.56 ± 10.84	22.79–34.34	15,585.00	33.5 ± 7.892	26.902–40.098	22–42	23.63 ± 11.56	13.96–33.29	15,585.00
**% Body Fat**	27.92 ± 7.50	23.92–31.91	14.24–39.94	32.113 ± 6.882	26.36–37.867	23.752–39.935	23.72 ± 5.74	18.92–28.51	14.24–31.1
**Fat Mass (kg)**	20.43 ± 7.50	16.43–24.43	8.83–32.91	21.11 ± 7.768	14.616–27.604	13.11–32.26	19.75 ± 7.70	13.31–26.18	8.83–32.91
**Fat-Free Mass (kg)**	52.02 ± 11.04	46.14–57.91	38.78–76.89	42.89 ± 3.414	40.036–45.744	38.78–48.54	61.15 ± 7.69	54.73–67.58	53.17–76.89
**HRrest (bpm)**	75.25 ± 9.13	70.39–80.11	62–93	73.75 ± 5.97	68.759–78.741	68–84	76.75 ± 11.73	66.94–86.56	62–93
**HRpost (bpm)**	120.69 ± 14.66	112.87–128.50	98–151	119.125 ± 8.254	112.225–126.025	108–131	122.25 ± 19.67	105.80–138.70	98–151
**HR1min (bpm)**	88.63 ± 13.29	81.55–95.71	67–118	89.875 ± 10.302	81.263–98.487	77–104	87.38 ± 16.39	73.68–101.08	67–118
**Ruffier Index**	8.46 ± 3.21	6.74–10.17	2.7–16	8.275 ± 2.196	6.439–10.111	5.7–11.8	8.64 ± 4.15	5.17–12.11	2.7–16
**Ruffier–Dickson Index**	7.74 ± 2.55	6.38–9.11	3.8–13.5	8.137 ± 1.678	6.734–9.541	6–10.6	7.35 ± 3.29	4.60–10.10	3.8–13.5
**Estimated VO_2max_ (mL·kg·min^−1^)**	28.57 ± 5.36	25.71–31.43	18.35–37.48	27.024 ± 4.439	23.312–30.735	18.35–32.01	30.12 ± 6.04	25.07–35.17	19.33–37.48
**Absolute VO_2max_ (L/min)**	2.05 ± 0.48	1.80–2.30	1.06–2.91	1.716 ± 0.311	1.456–1.976	1.06–2.01	2.38 ± 0.37	2.08–2.69	1.72–2.91

**Table A5:** Paired *t*-test results comparing baseline and after 8 weeks cycling.

Parameters	Total (*n* = 16)				Female (*n* = 8)				Male (*n* = 8)			
	t	*p*	Mean Difference	SE Difference	t	*p*	Mean Difference	SE Difference	t	*p*	Mean Difference	SE Difference
**Weight (kg)**	2.903	0.011	0.713	0.245	−1.46	0.188	−0.638	0.437	−3.068	0.018	−0.788	0.257
**BMI (kg/m^2^)**	2.678	0.017	0.248	0.092	−1.414	0.2	−0.24	0.17	−2.891	0.023	−0.255	0.088
**Waist (cm)**	2.588	0.021	0.987	0.382	−1.076	0.318	−0.537	0.5	−2.556	0.038	−1.438	0.563
**Hip (cm)**	0.093	0.927	0.069	0.739	0.933	0.382	0.55	0.589	−0.501	0.632	−0.688	1.372
**Waist/Hip ratio**	1.54	0.144	0.009	0.006	−1.214	0.264	−0.01	0.008	−0.918	0.389	−0.009	0.01
**Triceps (cm)**	1.225	0.239	0.844	0.689	−1.488	0.18	−0.625	0.42	−0.783	0.46	−1.063	1.358
**Chest (cm)**	0.098	0.923	0.063	0.636	−0.509	0.626	−0.25	0.491	0.103	0.921	0.125	1.217
**MidAxilla (cm)**	1.667	0.116	0.625	0.375	−2.049	0.08	−0.75	0.366	−0.734	0.487	−0.5	0.681
**Subscapula (cm)**	1	0.333	0.75	0.75	0	1	0	1.336	−0.18	0.862	−0.25	1.386
**Suprailiac (cm)**	−0.255	0.802	−0.125	0.491	−6.352	<0.001	−7	1.102	0	1	0	0.681
**Abdomen (cm)**	1.308	0.211	1.219	0.932	−0.033	0.974	−0.063	1.867	−1.198	0.27	−2	1.669
**Thigh (cm)**	2.913	0.011	1.438	0.493	−2.768	0.028	−2.188	0.79	−1.353	0.218	−0.688	0.508
**Body Fat (%)**	3.023	0.009	4.813	1.592	−2.034	0.081	−0.636	0.313	−1.472	0.184	−0.374	0.254
**Fat Mass (Kg)**	2.555	0.022	0.505	0.197	−2.242	0.06	−0.606	0.27	−2.135	0.07	−0.535	0.251
**Fat-Free Mass (Kg)**	−10.701	<0.001	−31.022	2.899	−0.111	0.914	−0.031	0.281	−0.921	0.388	−0.253	0.274
**HRrest (beats min^−1^)**	0.74	0.47	0.142	0.192	−2.324	0.053	−6.5	2.797	−1.498	0.178	−2.5	1.669
**HRpost (beats min^−1^)**	0.743	0.469	3.076	4.137	−2.642	0.033	−5.625	2.129	−2.238	0.06	−8.375	3.741
**HR1min (beats min^−1^)**	−2.485	0.025	−183.849	73.994	−1.088	0.313	−2.25	2.068	−7.159	<0.001	−9.25	1.292
**Ruffier Index**	2.718	0.016	4.5	1.656	−2.582	0.036	−1.437	0.557	−3.919	0.006	−2.013	0.514
**Ruffier–Dickson Index**	3.318	0.005	7	2.11	0.929	0.384	0.288	0.31	−4.546	0.003	−2.188	0.481
**EstimatedVO_2max_ (mL/kg/min)**	3.873	0.002	5.75	1.485	2.648	0.033	2.94	1.11	3.189	0.015	3.649	1.144
**AbsoluteVO_2max_ (L/min)**	4.621	<0.001	1.725	0.373	2.22	0.062	0.188	0.084	3.147	0.016	0.247	0.079

**Table A6:** Spearman’s correlation for females.

Variable		DF-Weight	DF-BMI	DF-WHR	DF-BF%	DF-RI	DF-RDI	DF-ESVO2	DF-ABVO2	Total Distance (m)
DF-Weight	Spearman’s rho	—								
	*p*-value	—								
DF-BMI	Spearman’s rho	0.994 ***	—							
	*p*-value	<0.001	—							
DF-WHR	Spearman’s rho	0.58	0.577	—						
	*p*-value	0.131	0.134	—						
DF-BF%	Spearman’s rho	0.275	0.31	0.086	—					
	*p*-value	0.509	0.462	0.84	—					
DF-RI	Spearman’s rho	0.319	0.371	0.031	0.731 *	—				
	*p*-value	0.441	0.365	0.942	0.04	—				
DF-RDI	Spearman’s rho	0.036	0.036	−0.111	0.539	−0.078	—			
	*p*-value	0.932	0.933	0.793	0.168	0.854	—			
DF-ESVO2	Spearman’s rho	−0.12	−0.048	−0.049	−0.095	−0.12	0.072	—		
	*p*-value	0.778	0.935	0.908	0.84	0.778	0.866	—		
DF-ABVO2	Spearman’s rho	0.072	0.143	0.049	0.143	0.084	0.144	0.952 **	—	
	*p*-value	0.866	0.752	0.908	0.752	0.844	0.734	0.001	—	
Total Distance (m)	Spearman’s rho	−0.491	−0.476	−0.282	−0.548	−0.551	−0.036	0.071	−0.214	—
	*p*-value	0.217	0.243	0.498	0.171	0.157	0.933	0.882	0.619	—

* *p* < 0.05, ** *p* < 0.01, *** *p* < 0.001.

**Table A7:** Spearman’s correlation for males.

Variable		DF-Weight	DF-BMI	DF-WHR	DF-BF%	DF-RI	DF-RDI	DF-ESVO2	DF-ABVO2	Total Distance (m)
1. DF-Weight	Spearman’s rho	—								
	*p*-value	—								
2. DF-BMI	Spearman’s rho	0.994 ***	—							
	*p*-value	<0.001	—							
3. DF-WHR	Spearman’s rho	0.239	0.218	—						
	*p*-value	0.568	0.603	—						
4. DF-BF%	Spearman’s rho	−0.109	−0.12	−0.315	—					
	*p*-value	0.797	0.776	0.448	—					
5. DF-RI	Spearman’s rho	−0.83 *	−0.867 **	−0.424	0.404	—				
	*p*-value	0.011	0.005	0.295	0.321	—				
6. DF-RDI	Spearman’s rho	−0.337	−0.371	−0.383	0.168	0.491	—			
	*p*-value	0.414	0.365	0.349	0.691	0.217	—			
7. DF-ESVO2	Spearman’s rho	−0.554	−0.587	0.038	−0.156	0.563	0.714	—		
	*p*-value	0.154	0.126	0.928	0.713	0.146	0.058	—		
8. DF-ABVO2	Spearman’s rho	−0.639	−0.659	0.038	−0.12	0.563	0.738 *	0.976 ***	—	
	*p*-value	0.088	0.076	0.928	0.778	0.146	0.046	<0.001	—	
9. Total Distance (m)	Spearman’s rho	0.349	0.287	0.179	−0.24	−0.036	−0.262	−0.071	−0.238	—
	*p*-value	0.396	0.49	0.672	0.568	0.933	0.536	0.882	0.582	—

* *p* < 0.05, ** *p* < 0.01, *** *p* < 0.001.

**Table A8:** Spearman’s correlation for age < 35years old.

Variable		DF-Weight	DF-BMI	DF-WHR	DF-BF%	DF-RI	DF-RDI	DF-ESVO2	DF-ABVO2	Total Distance (m)
DF-Weight	Spearman’s rho	0.982 ***	—							
	*p*-value	<0.001	—							
DF-BMI	Spearman’s rho	0.185	0.145	—						
	*p*-value	0.691	0.756	—						
DF-WHR	Spearman’s rho	−0.358	−0.396	0.037	—					
	*p*-value	0.431	0.379	0.938	—					
DF-BF%	Spearman’s rho	−0.844 *	−0.847 *	0.128	0.336	—				
	*p*-value	0.017	0.016	0.784	0.461	—				
DF-RI	Spearman’s rho	0.2	0.143	0.018	0.072	−0.505	—			
	*p*-value	0.667	0.783	0.969	0.878	0.248	—			
DF-RDI	Spearman’s rho	0.473	0.429	0.655	0.18	−0.054	0.071	—		
	*p*-value	0.284	0.354	0.111	0.699	0.908	0.906	—		
DF-ESVO2	Spearman’s rho	0.473	0.5	0.582	0.09	−0.09	−0.036	0.929 **	—	
	*p*-value	0.284	0.267	0.17	0.848	0.848	0.963	0.007	—	
DF-ABVO2	Spearman’s rho	0.182	0.071	0.218	−0.613	−0.18	0.321	−0.071	−0.286	—
	*p*-value	0.696	0.906	0.638	0.144	0.699	0.498	0.906	0.556	—
Total Distance (m)	Spearman’s rho	0.982 ***	—							
	*p*-value	<0.001	—							

* *p* < 0.05, ** *p* < 0.01, *** *p* < 0.001.

**Table A9:** Spearman’s correlation for age > 35 years old.

Variable		DF-Weight	DF-BMI	DF-WHR	DF-BF%	DF-RI	DF-RDI	DF-ESVO2	DF-ABVO2	Total Distance (m)
DF-Weight	Spearman’s rho	—								
	*p*-value	—								
DF-BMI	Spearman’s rho	0.998 ***	—							
	*p*-value	<0.001	—							
DF-WHR	Spearman’s rho	0.106	0.134	—						
	*p*-value	0.786	0.731	—						
DF-BF%	Spearman’s rho	0.56	0.546	−0.04	—					
	*p*-value	0.117	0.129	0.919	—					
DF-RI	Spearman’s rho	0.253	0.264	−0.232	0.331	—				
	*p*-value	0.511	0.492	0.548	0.384	—				
DF-RDI	Spearman’s rho	−0.026	−0.053	−0.537	0.166	0.696 *	—			
	*p*-value	0.948	0.892	0.136	0.669	0.037	—			
DF-ESVO2	Spearman’s rho	−0.69 *	−0.676 *	−0.246	−0.309	0.228	0.215	—		
	*p*-value	0.039	0.046	0.524	0.419	0.555	0.578	—		
DF-ABVO2	Spearman’s rho	−0.65	−0.636	−0.288	−0.286	0.252	0.245	0.997 ***	—	
	*p*-value	0.058	0.065	0.453	0.455	0.512	0.526	<0.001	—	
Total Distance (m)	Spearman’s rho	−0.201	−0.187	0.575	−0.214	−0.283	−0.51	0.184	0.133	—
	*p*-value	0.604	0.63	0.105	0.58	0.46	0.161	0.635	0.734	—

* *p* < 0.05, *** *p* < 0.001.
